# Liver respiratory-induced motion estimation using abdominal surface displacement as a surrogate: robotic phantom and clinical validation with varied correspondence models

**DOI:** 10.1007/s11548-024-03176-1

**Published:** 2024-05-29

**Authors:** Ana Cordón Avila, Momen Abayazid

**Affiliations:** Robotics and Mechatronics, Faculty of Electrical Engineering, Mathematics and Computer Science, University of Twente, 7500 AE Enschede, Netherlands

**Keywords:** Liver respiratory-induced motion, RGB-D camera, Correspondence model

## Abstract

****Purpose**:**

This work presents the implementation of an RGB-D camera as a surrogate signal for liver respiratory-induced motion estimation. This study aims to validate the feasibility of RGB-D cameras as a surrogate in a human subject experiment and to compare the performance of different correspondence models.

****Methods**:**

The proposed approach uses an RGB-D camera to compute an abdominal surface reconstruction and estimate the liver respiratory-induced motion. Two sets of validation experiments were conducted, first, using a robotic liver phantom and, secondly, performing a clinical study with human subjects. In the clinical study, three correspondence models were created changing the conditions of the learning-based model.

****Results**:**

The motion model for the robotic liver phantom displayed an error below 3 mm with a coefficient of determination above 90% for the different directions of motion. The clinical study presented errors of 4.5, 2.5, and 2.9 mm for the three different motion models with a coefficient of determination above 80% for all three cases.

****Conclusion**:**

RGB-D cameras are a promising method to accurately estimate the liver respiratory-induced motion. The internal motion can be estimated in a non-contact, noninvasive and flexible approach. Additionally, three training conditions for the correspondence model are studied to potentially mitigate intra- and inter-fraction motion.

## Introduction

Liver cancer is the third leading cause of cancer death [1]. According to the World Health Organization, it accounted for approximately 830.000 deaths in 2020. Percutaneous liver biopsy is the gold-standard technique implemented to evaluate the stage of the disease [2]. Furthermore, tumor ablation is the preferred treatment in single, and multiple hepatic tumors < 3 cm [3]. The success of percutaneous procedures depends on the accuracy and precision of needle placement.

Intra-fraction motion is a limiting factor that affects the outcome of hepatic procedures. This motion corresponds to the involuntary movements of the patient (such as respiration) [4]. Managing respiration in abdominal percutaneous procedures is essential to achieve accurate needle placement. Inaccurate insertion can lead to misdiagnosis and incomplete treatment [4, 5].

Currently, breath-holding techniques are implemented to overcome the respiratory-induced motion (RIM) in hepatic percutaneous procedures [6]. The clinician has limited time to insert the needle, while the patient performs a breath-hold. Some patients cannot hold their breath for sufficient time which increases the complexity and duration of the procedure [6].

The direct use of imaging modalities to track the tumor motion comes with its complications as out-of-plane tumor motion will not be directly tracked. Additionally, constant monitoring will expose the patient and clinician to excessive ionizing radiation (e.g., CT). Alternatively, advanced tracking techniques are developed to estimate the tumor motion in real time.

Motion estimation based on surrogate signals offers the possibility to accurately track in real time the tumor. A correspondence model is trained to establish the relationship between RIM and the surrogate signal [7]. Surrogates are external signals that provide highly correlated indirect measurements of the internal motion at a high-frame rate [7]. Examples of surrogate signals are optical markers, reference needles and electromagnetic tracker systems [5, 8–11]. Motion compensation techniques are well-developed and established in the field of radiation therapy. These can be adapted to needle interventions. An example of a radiation therapy motion estimation system is Synchrony (Accuracy Incorporated, Sunnyvale, CA) that is integrated into CyberKnife (Accuracy Incorporated). This system uses external markers as surrogates to estimate and robotically compensate for the RIM [12, 13].

Furthermore, different systems have been developed for marker-less optical surface imaging (OSI). These systems offer the possibility of relying on more points than marker-based techniques [14]. Examples of such systems are AlignRT (VisionRT Ltd, London, UK) and IDENTIFY (Varian Medical System Inc., Palo Alto, CA). These systems have been proposed as alternative methods in radiation therapy to indirectly track the RIM using the abdominal surface changes. Some researchers have extended the application of these systems to estimate internal breathing parameters or internal motion [15–17]. Future lines of research for OSI systems consider their implementation for respiratory-induced tumor motion prediction [14]. Needle insertion procedures can benefit from OSI technology as the clinician’s hand, and needle can occlude markers partially (unlike radiation therapy) and thus surface scans with more points is expected to be more reliable.

In [18], the current research status on respiratory motion for abdominal/thoracic tumor treatment is reviewed. It is highlighted that future lines of research should move toward non-contact methods. Examples of non-contact methods are marker-less approaches using RGB-D cameras to extract the respiratory motion [19–22]. Similarly to OSI methods, the use of RGB-D cameras as surrogate offers the opportunity to obtain non-contact observations of numerous abdominal points. However, RGB-D cameras are consumer-grade products that are flexible, portable and inexpensive. These advantages highlight their potential as surrogate signals. Previous studies on RGB-D cameras did not include validation on human subjects to estimate the internal RIM [19–22]. Thus, the subsequent stage should incorporate human subject validation into the study of RGB-D cameras as surrogate signals.

This study aims to create human subject-specific motion models using an abdominal surface displacement measured using an RGB-D camera as a surrogate signal. The created models estimate the liver RIM. Two sets of validation experiments were conducted, first, using a robotic liver phantom and, secondly, performing a clinical study on human subjects. Additionally, in the clinical study, we aim to investigate how different training sessions can impact the accuracy of motion models. The results of this study introduce RGB-D cameras as a surrogate signal that is noninvasive, contactless and inexpensive, and present the impact of different training conditions on the accuracy of hepatic RIM models.

## Materials and methods

This study aims to create a respiratory motion model using an RGB-D camera as a surrogate signal.

**Fig. 1:**
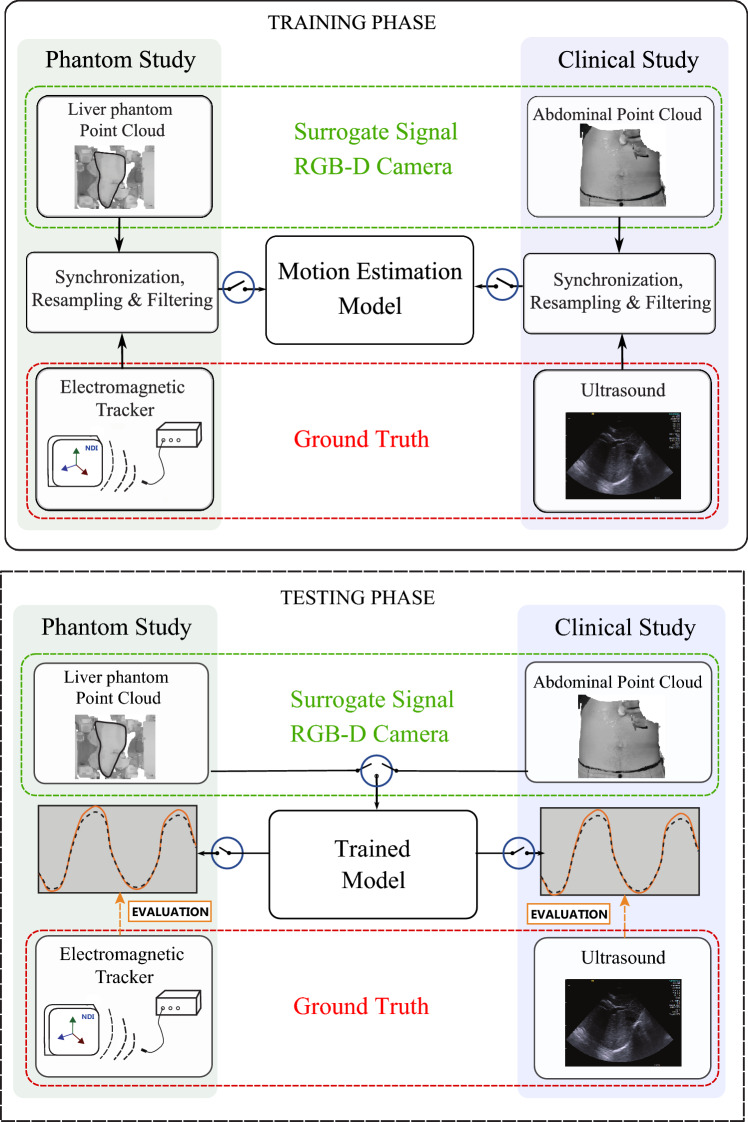
Motion models can be divided in two main phases, training and test. Point clouds of the liver phantom or the abdominal region were obtained alongside with the ground truth. The performance of the correspondence model was evaluated using performance metrics

The study was validated conducting a robotic liver phantom experiment and in a clinical study. An electromagnetic sensor was placed inside the liver phantom, and ultrasound was used to extract the liver motion of the participants. These signals corresponded to the ground truth of the models. A correspondence model was trained to establish the relationship between the surrogate signal and the ground truth. Motion models were studied for the anterior–posterior (AP) and superior–inferior (SI) directions of motion in the liver phantom experiments, and for the SI motion in the clinical study since organ motion is mainly in this direction [4]. The performance of the models was evaluated using the ground truth (Fig. 1).

**Fig. 2:**
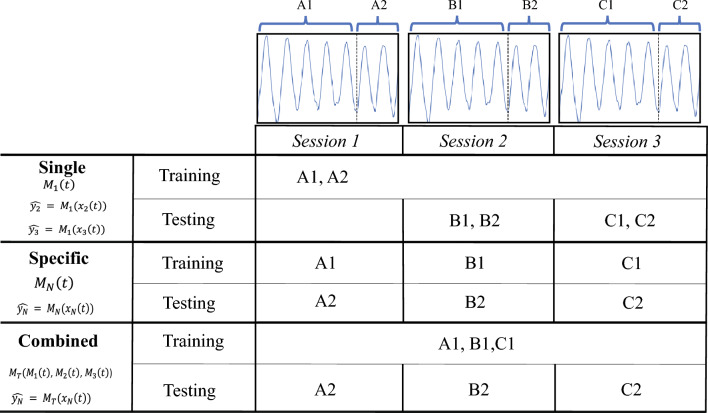
Three models were created: single, specific, and combined. The single model is trained using data from the first session and is tested using the data of the remaining sessions. The specific model is trained and tested with data from the same session. The combined model uses part of the data from all sessions for the training and is tested with the remaining part

### RGB-D as surrogate signal

The RGB-D camera Intel RealSense D435i (Santa Clara, California, United States) was selected. The camera has a sampling rate of 30 Hz, and depth frames were aligned with RGB frames. Point clouds of the skin of the abdomen or the liver phantom were obtained from the RGB and depth frames. The point cloud that corresponded to the maximum inhalation was selected as reference point cloud. The mean difference between each point in a point cloud and its closest point in a reference point cloud served as a surrogate.

### Correspondence models

Regression models are accurate and feasible candidates for estimating respiratory motion [8, 11]. In the present work, a polynomial regression was applied to define the relationship between the motion data and the surrogate.

1 $$\begin{aligned} {y_i \approx y_i(\beta ) = \beta _0 + \beta _1x + \beta _2x^2+ ... +\beta _nx^n} \end{aligned}$$

such that ${\beta =[\beta _0,\beta _1, ..., \beta _n]}$ are the parameters obtained during training, *x* represents the surrogate, $y_i$ is the ground truth, and $y_i(\beta )$ the estimated liver displacement. The ordinary least squares (OLS) method was applied to obtain the $\beta$ coefficients that define best the relationship between surrogate and ground truth. The OLS is based on minimizing a cost function (${J(\beta )}$) using the training sets (Eq. 2).

2 $$\begin{aligned} {J(\beta ) = \sum _{i=1}^{N} (y_i - y_i (\beta ))^2} \end{aligned}$$

For the clinical study, we propose three approaches to compute the $\beta$ parameters of the model (Fig. 2). Each subject participated in three sessions $S_{n=1,2,3}$ containing their corresponding surrogate and ground truth data (*x*, *y*).

- *Single Model*: A motion model $M_1$ is created using (*x*, *y*) from the first session $S_{1}$ for training and the data from the other sessions $S_{2}$ and $S_{3}$ for testing.
- *Specific Model*: A specific model $M_{n}$ for a session *n* is trained using $70\%$ of (*x*, *y*) in $S_{n}$ and tested using the remaining $30\%$.
- *Combined Model*: This approach creates a motion model $M_T$ using $70\%$ of (*x*, *y*) in $S_{1}$, $S_{2}$, and $S_{3}$ combined for training and $30\%$ for testing.

Once the ${\beta }$ parameters are computed, new data can be tested by applying Eq. 1. In the liver phantom experiments, only the specific model was implemented because the simulated respiratory motion follows a periodic pattern. Finally, the evaluation of the performance of the regression models was obtained by calculating the mean absolute error (MAE), and the coefficient of determination.

### Ground truth: Motion tracking

#### Electromagnetic tracking

An electromagnetic (EM) sensor (Northern Digital Inc., Waterloo, Canada) was inserted into the liver phantom to simulate the moving tumor. The OpenIGT (Open Network Interface for Image-Guided Therapy) was installed to acquire the data from the sensor [23]. The EM tracker has a sampling rate of 40 Hz, and it was synchronized to the surrogate data through timestamps.

#### Ultrasound imaging

**Fig. 3:**
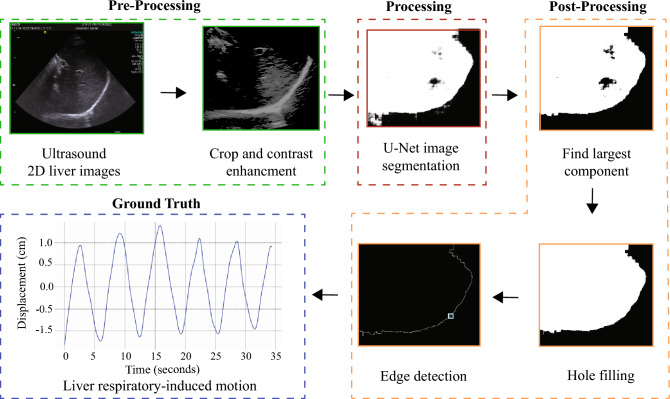
Obtained raw ultrasound images are pre-processed, segmented using a neural network, and post-processed to extract the 1D respiratory-induced motion of the liver

Different processing steps were implemented to acquire the 1D displacement of the liver (Fig. 3). Pre-processing steps consisted in enhancing the contrast and manually cropping the images to have a size of $512\times 512$ pixels^2^. A U-Net network was implemented to segment the liver in the ultrasound images and post-processing steps involved the detection of the largest detected component, and the implementation of morphological operators.

A U-Net convolutional neural network was created for each subject. A set of manually created masks of the liver served as ground truth. MobileNetV2 model was implemented in the encoder structure to reduce the number of trainable parameters. Once the network had been trained, it was implemented to segment the remaining images from the ultrasound recordings.

Post-processing steps included identifying the largest connected component in the binary image, filling the holes of the detected component, and extracting its edges. The resulting segmented images and the quality of the detected edges were evaluated by extracting the Dice score and Hausdorff distance of 20 randomly selected images from each recording. The liver motion was extracted by computing the displacement in the *x*-axis of the obtained edges. The displacement of the edges was converted into cm. It was observed that 1 pixel corresponded to 0.027 cm. The motion represented the SI hepatic RIM.

### Validation

#### Liver phantom experiments

**Fig. 4:**
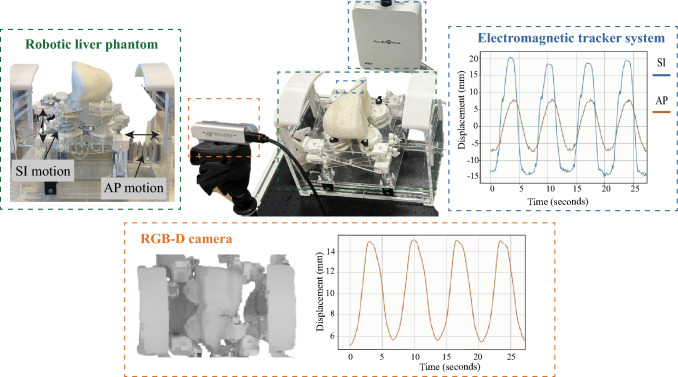
Robotized liver phantom (green), RGB-D camera was used as surrogate signal (orange) and an electromagnetic system (blue) simulated the tumor

A modified version of the robotic liver phantom presented by Naghibi et al. [24] was used (Fig. 4). The liver phantom consists of two parts, a soft tissue that simulates the liver, and a cart that includes actuators to move the liver mimicking respiration. The soft tissue is made of a silicon resin mixed with styrofoam beads. The cart is actuated pneumatically, and has an amplitude of motion of 30 mm in *x*- and 10 mm in the *y*-direction, simulating the SI and AP motion, respectively. The simulated breathing frequency corresponds to 0.25 Hz. The latest version of the liver phantom includes four pneumatic actuators for the AP direction, and one for the SI. The control of the motion is achieved using an Arduino Uno microcontroller (Arduino, Tornio, Italy).

#### Clinical study

**Fig. 5:**
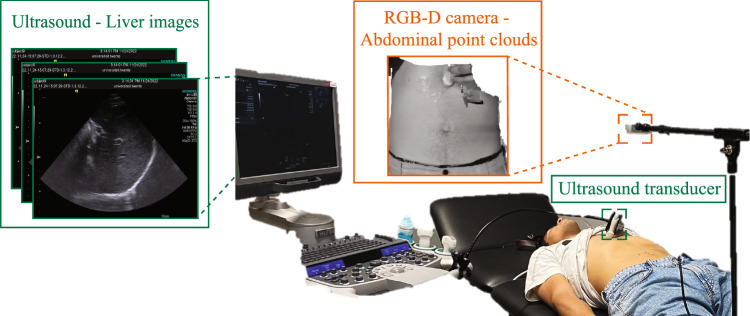
Liver ultrasound images are extracted as ground truth (green), and the RGB-D (orange) was used as surrogate signal

Six healthy human subjects (three males and three females) participated in this study. All subjects signed a consent form. Participants were asked to breath for 1 min and perform a breath-hold at the beginning and end of the experiment. Experiments were repeated three times per participant. Ultrasound liver images and RGB-D frames of the abdomen were acquired (Fig. 5). Signal synchronization was achieved using the breath-holds in between each breathing session. No phase-shift between internal and external motion was considered since it is has been shown to be negligible for the liver [25].

Ultrasound ACUSON S2000 (HELX Evolution, Siemens Healthineers, Erlangen, Germany) was used. The frequency was set to 3–4 MHz, and the depth at 18 cm. Ultrasound images had a size of $768\times 1024$ pixels^2^ and a sampling frequency of 15 Hz.

## Results

### Liver phantom experiments

**Fig. 6:**
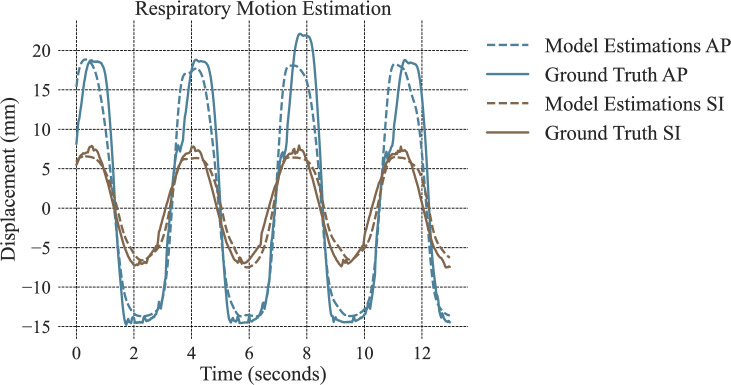
Liver phantom motion model estimations (dashed lines) and ground truth (solid lines) data from the test set for the AP (orange) and SI (blue) directions of motion

The results of the liver phantom models are presented in Fig. 6 and Table 1. Two motion models were created, one for the SI direction and a second model for the AP direction. It can be observed that all errors are below 3 mm, and the coefficients of determination are above 90$\%$.

**Table 1:** Liver phantom motion model estimations error mean absolute error values are in mm

Performance	Motion direction		
	Metrics	Superior–inferior	Anterior–posterior
Train	MAE	$2.6 \pm 0.2$	$1.0 \pm 0.2$
	${R^2}$	$92.2 \pm 1.1 \%$	$94.6 \pm 2.3$
Test	MAE	$2.8 \pm 0.3$	$0.8 \pm 0.2$
	${R^2}$	$91.8 \pm 2.4 \%$	$96.0 \% \pm 1.9$

**Table 2:** Liver segmentation evaluation using the Dice score and the Hausdorff distance

Subject	Session	Segmentation metrics		
		Dice score	Hausdorff distance	Hausdorff distance ROI
1	1	$97.9 \pm 0.6\%$	$13.0\pm 4.6$	$1.6 \pm 1.0$
	2	$97.2 \pm 1.9\%$	$12.6\pm 5.0$	$1.5\pm 0.7$
	3	$95.8 \pm 2.8\%$	$14.5 \pm 6.0$	$2.0\pm 1.8$
2	1	$97.9 \pm 1.0\%$	$10.5\pm 5.0$	$1.8\pm 0.8$
	2	$95.5 \pm 2.7\%$	$13.2\pm 4.9$	$3.1\pm 3.4$
	3	$95.0 \pm 3.7\%$	$13.8\pm 6.9$	$3.5\pm 6.9$
3	1	$97.9 \pm 1.2\%$	$7.9\pm 3.0$	$2.3\pm 1.7$
	2	$95.0 \pm 3.1\%$	$16.6\pm 11.0$	$9.7\pm 10.3$
	3	$92.9 \pm 3.3\%$	$24.8\pm 8.6$	$6.3\pm 6.0$
4	1	$80.4 \pm 13.0\%$	$30.0\pm 17.1$	$7.7\pm 6.1$
	2	$92.9 \pm 4.0\%$	$14.7\pm 4.4$	$3.5\pm 2.6$
	3	$86.7 \pm 4.3\%$	$27.4\pm 7.9$	$20.5\pm 13.3$
5	1	$90.0 \pm 3.1\%$	$19.3\pm 4.0$	$1.8\pm 1.4$
	2	$91.2 \pm 2.9\%$	$20.1\pm 3.7$	$1.8\pm 1.0$
	3	$95.1 \pm 2.2\%$	$16.7\pm 7.3$	$3.0\pm 1.9$
6	1	$93.1 \pm 5.9\%$	$16.5\pm 7.6$	$4.8\pm 4.1$
	2	$96.6 \pm 1.1\%$	$9.6\pm 2.9$	$3.1\pm 1.3$
	3	$95.5 \pm 2.5\%$	$15.6\pm 7.6$	$4.1\pm 4.4$

Hausdorff distance in mm

### Clinical study

Ultrasound images were segmented to track the liver RIM. The tracked motion represents the ground truth. The ground truth quality was evaluated using segmentation metrics (Table 2). Finally, three different models were created.

#### Ultrasound liver segmentation

Liver segmentation was evaluated by computing three metrics. The Dice score, the Hausdorff distance and the Hausdorff distance of a specific region of interest (ROI) were calculated. The selected ROI encapsulated the borders of the liver which move due to the respiratory motion and were used for tracking. The resulting Dice score is above 80$\%$ for all cases. A low Hausdorff distance in the ROI indicates that the method applied to the images is accurately detecting the edges allowing the extraction of the ground truth. The Hausdorff distance error is mitigated by filtering the final 1D signal obtained during edge tracking.

**Fig. 7:**
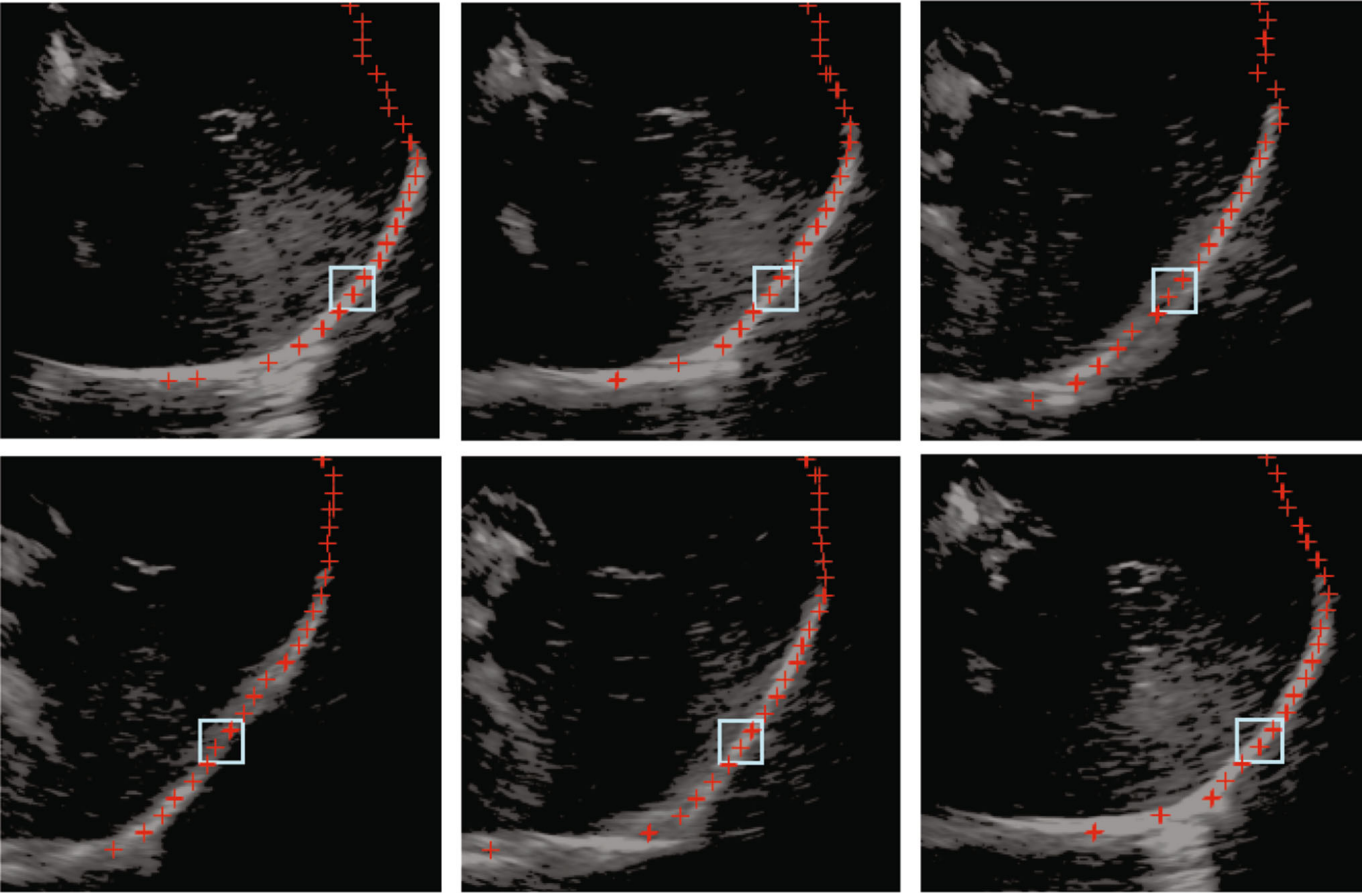
Ultrasound images of the liver with segmentation results (depicted in red). The ROI selected is represented by the blue box

Figure 7 presents a set of ultrasound images of the liver of the same subject within the same session. It can be observed the edges of the masks obtained after segmentation and the selected ROI.

#### Motion data analysis

Table 3 displays the liver RIM observed in all participants. The motion represents the overall peak-to-trough distance for each session. The liver displays a total mean displacement of $35.2 \pm 12.2$ mm.

**Table 3:** Liver displacement over each session for each subject. All units are in mm

	Liver motion ($\mu \pm \sigma$)		
Subjects	Session 1	Session 2	Session 3
1	33.0 ± 7.9	27.5 ± 4.1	19.2 ± 0.6
2	22.1 ± 2.7	19.7 ± 2.0	16.8 ± 2.9
3	25.4 ± 5.5	29.1 ± 5.0	26.1 ± 4.8
4	44.6 ± 4.8	48.2 ± 5.8	38.5 ± 5.1
5	49.4 ± 3.4	45.9 ± 4.1	55.6 ± 6.5
6	46.9 ± 4.9	44.9 ± 3.6	37.5 ± 2.1

#### Motion models

**Fig. 8:**
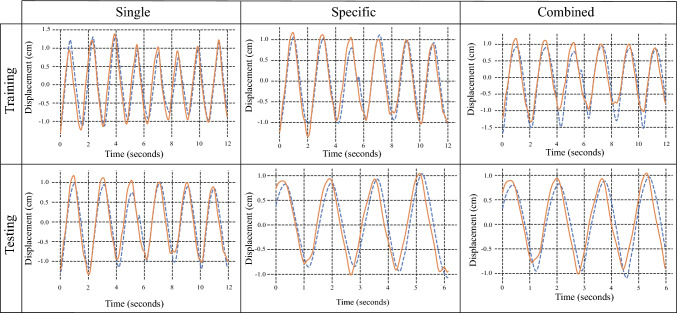
Motion model results for subject 2. The dashed blue lines correspond to the model estimations, and the solid orange lines represent the ground truth

Table 4 presents the results of the single, specific and combined models. Figure 8 displays the performance of each model for subject 2.

## Discussion

This work introduces the use of RGB-D cameras as a surrogate signal to estimate the liver RIM in a human subject study. This surrogate signal offers a non-contact and flexible approach to address the RIM during the treatment and diagnosis of hepatic tumors. The obtained results show that the RGB-D camera can be used to estimate the liver RIM with errors below 3 mm for the liver phantom experiments. In the human subject study, errors below 3 mm were achieved when data from the same session is included during training. However, relying on a model that was trained using data from a different session leads to higher estimation errors.

Furthermore, we aim to translate our presented work into the challenging environment of liver percutaneous procedures. The EPIONE robotic system (Quantum Surgical, Montpellier, France) is a novel commercially available system used to perform liver tumor ablation. This system tracks abdominal motion using optical markers attached at the patient’s abdomen. The abdominal motion is used to compensate for the RIM by allowing insertion only when the target is at the expected position but it does not estimate the tumor location. Unlike during radiation therapy, during needle insertion physicians and medical staff need to be in close proximity to the patient. This introduces complexity and the potential for occlusion of the optical markers. The proposed work on RGB-D cameras could compensate for this limitation since the present method relies on numerous abdominal points.

**Table 4:** Overall performance for all subjects for the proposed motion models (single, specific, and combined). Mean absolute error values are in mm

Subject	Performance metrics	Motion models		
		Single	Specific	Combined
1	Train MAE	2.6	$1.9\pm 0.8$	$2.0\pm 0.5$
	${R^2}$	$92.9 \%$	$94.4\pm 2.2 \%$	$88.2\pm 3.9 \%$
	Test MAE	$6.1\pm 0.5$	$2.1\pm 1.1$	$1.89\pm 0.72$
	${R^2}$	$65.6\pm 2.3 \%$	$87.0\pm 11.2 \%$	$87.0\pm 7.8\%$
2	Train MAE	1.7	$1.7\pm 0.4$	$2.0\pm 0.5$
	${R^2}$	$91.3 \%$	$91.3\pm 1.9 \%$	$88.2\pm 7.2 \%$
	Test MAE	$1.9\pm 0.1$	$1.7 \pm 0.7$	$1.9 \pm 0.7$
	${R^2}$	$87.7\pm 1.3 \%$	$88.5\pm 7.1 \%$	$87.0\pm 7.8 \%$
3	Train MAE	2.5	$2.3\pm 0.2$	$3.1\pm 0.8$
	${R^2}$	$86.5 \%$	$90.1 \pm 1.0 \%$	$79.3\pm 1.4 \%$
	Test MAE	$3.0\pm 0.2$	$2.3\pm 1.2$	$2.3\pm 0.8$
	${R^2}$	$85.1\pm 1.3$	$90.7\pm 8.5 \%$	$91.7\pm 4.4 \%$
4	Train MAE	1.9	$3.5\pm 1.3$	$4.7\pm 1.7$
	${R^2}$	$97.7 \%$	$90.5\pm 3.8 \%$	$83.5\pm 2.1 \%$
	Test MAE	$5.4\pm 0.1$	$2.9\pm 0.4$	$3.6\pm 0.2$
	${R^2}$	$81.1\pm 2.8 \%$	$93.2\pm 2.3 \%$	$91.1\pm 3.3 \%$
5	Train MAE	2.86	$3.4\pm 0.2$	$4.0 \pm 0.1$
	${R^2}$	$95.0 \%$	$94.3\pm 1.0\%$	$91.2 \pm 4.0 \%$
	Test MAE	$8.1\pm 5.2$	$2.0 \pm 0.3$	$4.3\pm 2.6$
	${R^2}$	$69.0\pm 25.8 \%$	$97.9\pm 0.2\%$	$91.3\pm 7.6\%$
6	Train MAE	2.7	$2.7\pm 0.6$	$3.8\pm 1.3$
	${R^2}$	$94.5\%$	$94.2 \pm 1.5\%$	$88.4 \pm 5.1 \%$
	Test MAE	$3.9\pm 0.6$	$3.6\pm 0.6$	$3.5\pm 0.4$
	${R^2}$	$86.3\pm 2.5 \%$	$88.4\pm 1.5\%$	$89.0\pm 2.1\%$
Overall	Train MAE	$2.3\pm 0.5$	$2.7\pm 0.7$	$3.3\pm 1.0$
	${R^2}$	$93.0\pm 3.8$	$92.1\pm 1.8$	$86.1\pm 4.2 \%$
	Test MAE	$4.5\pm 2.1$	$2.5\pm 0.7$	$2.9\pm 0.9$
	${R^2}$	$82.0\pm 7.6$	$91.7\pm 3.5$	$89.5 \pm 2.0\%$

Finally, motion in the AP and lateral directions, and other sources of motion (such as the heartbeat) were not considered in this study. Therefore, a more realistic model should incorporate all directions and sources of motion. Additionally, in percutaneous procedures, the tool-tissue interaction during needle insertion can cause a deformation in the tissue which could move the tumor. Motion models should incorporate such interactions for an accurate estimation of the location of the tumor.

## Conclusion

In this study, an RGB-D camera was implemented to estimate the hepatic RIM. Two sets of validation experiments were performed (liver phantom experiments and clinical study). The results show that the RGB-D camera can be used to accurately estimate the RIM in the two scenarios. Additionally, variations in training sets affects the accuracy of motion models. Specific and combined models showed the highest performance indicating that motion model training can perform better when incorporating data from the current session. However, the results in the single model indicated that intra-fraction motion is more difficult to model by relying only on data from previous sessions.
